# A review on the clinical implementation of respiratory-gated radiation therapy

**DOI:** 10.2349/biij.3.1.e40

**Published:** 2007-01-01

**Authors:** CB Saw, E Brandner, R Selvaraj, H Chen, M Saiful Huq, DE Heron

**Affiliations:** University of Pittsburgh Cancer Institute, Pittsburgh, Pennsylvania, United States

**Keywords:** respiratory motion, motion-gated technique, radiation therapy

## Abstract

Respiratory-gated treatment techniques have been introduced into the radiation oncology practice to manage target or organ motions. This paper will review the implementation of this type of gated treatment technique where the respiratory cycle is determined using an external marker. The external marker device is placed on the abdominal region between the xyphoid process and the umbilicus of the patient. An infrared camera tracks the motion of the marker to generate a surrogate for the respiratory cycle. The relationship, if any, between the respiratory cycle and the movement of the target can be complex. The four-dimensional computed tomography (4DCT) scanner is used to identify this motion for those patients that meet three requirements for the successful implementation of respiratory-gated treatment technique for radiation therapy. These requirements are (a) the respiratory cycle must be periodic and maintained during treatment, (b) the movement of the target must be related to the respiratory cycle, and (c) the gating window can be set sufficiently large to minimise the overall treatment time or increase the duty cycle and yet small enough to be within the gate. If the respiratory-gated treatment technique is employed, the end-expiration image set is typically used for treatment planning purposes because this image set represents the phase of the respiratory cycle where the anatomical movement is often the least for the longest time. Contouring should account for tumour residual motion, setup uncertainty, and also allow for deviation from the expected respiratory cycle during treatment. Respiratory-gated intensity-modulated radiation therapy (IMRT) treatment plans must also be validated prior to treatment. Quality assurance should be performed to check for positional changes and the output in association with the motion-gated technique. To avoid potential treatment errors, radiation therapist (radiographer) should be regularly in-serviced and made aware of the need to invoke the gating feature when prescribed for selected patients.

## INTRODUCTION

Conformal radiation therapy (CRT) and intensity-modulated radiation therapy (IMRT) treatment techniques require a higher level of accuracy in patient setup compared to other traditional treatment techniques. This is because CRT requires the leaves of the multileaf collimation (MLC) system to conform to the shape of the target area defined by the beam-eye-view projection while IMRT modulates the intensity across each treatment field per gantry angle based on the target shape in three dimensions [1-3]. Any unintended deviation in patient setup or target motion could lead to having the target outside and/or critical structures inside the treatment fields resulting in inadequate dose to the target and exposure to critical structures. Because of the need for higher accuracy, patients are now set up using on-board imaging systems referred to as image-guided radiation therapy (IGRT) [4-5]. Even with the implementation of IGRT techniques, residual intrafraction motion cannot be completely eliminated and hence has become an important issue for treatments performed with CRT and IMRT, which utilize smaller margins. Respiratory, skeletal muscular, cardiac, gastrointestinal systems and other physiologic processes can cause intrafraction motion. These motions are very natural and are unpredictable to an extent except for respiratory motion. The impact of respiratory motion on targets and organs has been reported by a number of investigators. Thoracic tumours have been observed to move by more than 2 cm [6]. The movement of lung lesions has been found to be greatest in the lower lobes [7]. Likewise, the pancreas and kidneys can move by more than 2 cm while livers can move by more than 3 cm. [8-9]. Without the means to limit respiration-induced target or organ motion, large treatment fields have to be used resulting in the irradiation of surrounding normal tissues; and hence increasing the risk of complications. Conversely, if smaller treatment fields are used, the target may move out of the treatment field resulting in an underdose or margin of miss of the target.

Some techniques such as breath-holding, forced-shallow breathing and respiratory-gated treatment techniques have been implemented to account for respiratory motion. In the breath-holding treatment technique, the patient is asked to hold his/her breath during the course of treatment [10]. This treatment technique is especially difficult for patients having compromised pulmonary function, which is the case for most of the lung cancer patients. In the forced-shallow breathing treatment technique, a physical plate is placed and fixed over the abdominal region to restrict breathing motion and thereby limits the excursion of targets and organs [11]. The disadvantage of these mechanisms is it may cause great patient discomfort. An alternate to the normal breathing obstruction methods of treatment is the use of the respiratory-gated treatment technique. The implementation of the respiratory-gated treatment technique requires some form of marker to generate the respiratory cycle on which gating window can be set to turn the beam ON within and OFF outside the gate. Two types of internal markers are being developed at Hokkaido University [12-13] while an external marker is being developed by a commercial vendor. The focus of this paper is to review the implementation of the respiratory-gated treatment technique based on an external marker as a surrogate signal for target motion.

## METHODS AND MATERIALS

The development of respiratory-gated radiation therapy has been somewhat rapid, hence a clear understanding of the potential uses and limitations are important. The respiratory-gated treatment technique assumes that the respiratory motion is periodic and the target moves in a similar periodic fashion. The radiation beam is turned ON when the target moves into the treatment field as planned. Mechanisms have been introduced to synchronise energising of the radiation beam to the respiratory cycle. When using an external marker as described below, it should be noted that the radiation is synchronized to turn ON based on the respiratory cycle and not the motion or position of the target. Much of the data in this review has been derived from articles published in one of the four special issues on image-guided radiation therapy (IGRT) of Medical Dosimetry Journal by Brandner *et al* [14], Jiang [15], Keall *et al* [16], and Saw *et al*. [5] in 2006. In addition, the experience of implementing the respiratory-gated treatment technique based on an external marker at this author’s institution is incorporated into this manuscript.

**Figure 1:**
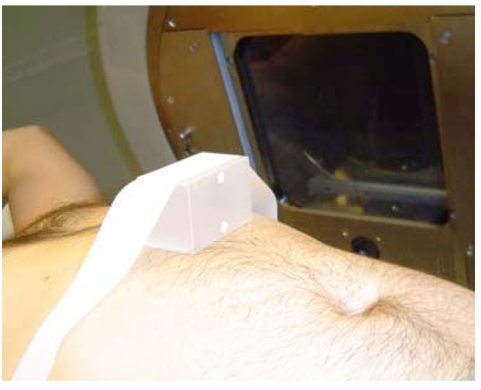
The placement of a marker block below the xyphoid process to generate respiratory signal. [With permission from C.B.Saw Publishing].

**Figure 2:**
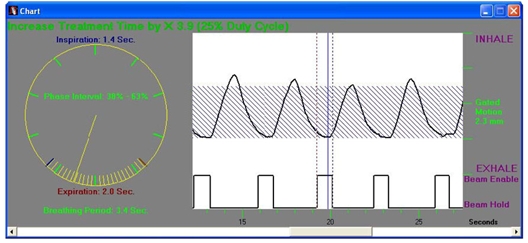
The marker block signal as a function of time from the RPM system.

Although various vendors are introducing respiratory-gated devices, the most widely discussed is the Varian Real-time Position Management (RPM) system. A passive marker block is placed on the abdominal region between the xyphoid process and umbilicus as shown in Figure 1. The position of placement must be carefully chosen to maximize the amplitude of the marker motion on the patient. The marker block is made of plastic with two rounded metallic reflectors (separated by 3 cm for calibrating the amplitude) placed on one side. An infrared camera, mounted at the foot of the CT table for 4DCT scanning and also at the foot of the treatment couch for treatment views, tracks the movement of the markers and hence produces the respiratory cycles as shown in Figure 2. The display on the computer monitor running the RPM software includes an animated bar travelling up and down following the motion of the marker block on the patient as a function of time. A duplicate display is placed in the room for the patient to visualise and control the amplitude of his/her respiration. The bar travels on a blue background, which represents the full range of the block motion identified over the patient’s first few respiratory cycles recorded by the RPM system. When the patient’s respiratory motion drives the signal beyond the range, the bar changes colour indicating to the patient that he/she should reduce inhaling or exhaling. In this manner, the patient can monitor the amplitude of his/her respiration and controls the response. In addition, audio coaching can also be instituted to improve the periodicity of the respiration motion. The audio coach instructs the patient to “breathe-in” and/or “breathe out” at regular intervals. The time between each instruction is controlled by the user and can be programmed to match the patient’s normal respiratory cycle.

The gating window is set based on the respiratory signal presented in Figure 2. The maximum vertical movement of the marker block represents the amplitude of the signal. The upper portion of the signal represents the inhaled phase while the lower portion of the signal is the exhaled phase. The distance in time between repeated points on the respiratory cycle represents a complete period and is divided into a number of phases of equal duration. Although the respiratory signal shown in Figure 2 exhibits excellent periodicity, there are some cases where the amplitude decays or suddenly changes, or periodicity changes, reflecting the status of the patient. A periodicity filter within the software can be used to hold the beam off during such sudden irregularities. The gating window is set in two ways referred to as phase gating and amplitude gating. In phase gating as shown in the figure, the beam “ON” is triggered at a certain point past the maximum inhaled state and thereafter on the onset of inhaling process to inhibit the beam. The gating window is typically set when the patient is in the exhaled state where the target moves the least. The range in amplitude is used to set the gating window in amplitude gating. At present, investigation is ongoing as to whether phase gating or amplitude gating is optimal for the treatment.

As stated earlier, the movement of the surrogate signal may not be directly correlated to the movement of the tumour or target. Hence, an assessment of the correlation must be carried out by performing 4DCT scans. There are two types of 4DCT scans identified as prospective and retrospective 4DCT scans. In the prospective 4DCT scanning technique, the respiratory-gated CT scanner collects images only at one phase or a limited portion of a patient respiratory cycle. Then the couch is moved to the next indexed position. The process is repeated to obtain one volumetric CT image collected at a specific phase of the respiratory cycle. In the retrospective 4DCT scanning technique, the respiratory-gated CT scanner acquires images continuously during all phases while the couch remains temporarily stationary. Each acquired image is identified by the phase and also the couch index position. The couch then moves to the next indexed position and the continuous acquisition is repeated until the volume of interest has been scanned. Each phase can then be separately reconstructed. The outcome of retrospective 4DCT technique is the acquisition of multiple volumetric CT images, each representing one phase of the respiratory cycle.

**Figure 3:**
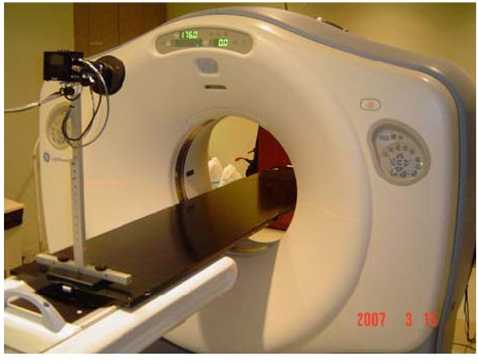
The location of the infrared camera at the foot of the couch for tracking the marker block in the RPM system.

At the University of Pittsburgh Medical Center (UPMC) Cancer Centers and the University of Pittsburgh Cancer Institute, the RPM system integrated into the GE Lightspeed CT-simulator as shown in Figure 3 is used to perform the retrospective 4DCT scans. Initially, the radiation oncologist identifies patients for whom motion is of concern. However, the patient must be assessed to have a periodic respiratory cycle and uncompromised pulmonary function, and is stable. Once the patient arrives at the medical centre, the procedure of performing the 4DCT scan is described to the patient. Patient positioning is instituted by designing the patient support system, by arranging the immobilisation devices, and by using treatment aids. After the patient positioning procedure is complete, a marker block is placed on the patient abdominal region and the RPM system is activated. The CT technologist or radiation oncology physicist explains and instructs the patient to perform the breathing sequence in response to the audio coaching instructions of “breathe in” followed by an exhale in a manner that the patient feels comfortable with, between each instruction.

An initial scout view and a helical CT scan through the volume of interest are acquired, and at the same time, the marker motion is monitored to identify the respiratory cycle and also the inhalation and exhalation times. Next, the tracing of the respiratory cycle is evaluated with audio coaching implemented to set up the system. The longest observed time is entered into the audio coaching configuration since most patients breathe more deeply in response to coaching. The mono-phasic audio coaching (i.e. “breathe in”) is activated and the patient is instructed to follow the coaching instructions. The rhythm of the coaching instruction is adjusted until the patient is comfortable with the pace. Once the audio coaching pace is established, the CT-simulator is programmed to acquire in the cine acquisition mode that is to remain at each couch location for the entire respiratory cycle plus one second and to acquire an image in a fraction (typically one-tenth) of the respiratory cycle. This allows the CT-simulator to acquire multiple images throughout the respiratory cycle at the same couch location. A slice thickness of 2.5 mm is commonly used for 4DCT scans at this author’s institution. For example, if images are acquired every 0.5 seconds for a 5-second breathing cycle, it results in 10 images representing 10 different phases of the respiratory cycle. With the programmed time of one additional second, 12 images are acquired instead of 10 images, two of which are extra images. The rationale for extending an additional second is to accommodate slight changes in the patient’s respiratory cycle. When all the images at one couch location have been acquired, the radiation beam is turned off and the couch automatically moves to the next indexed position and the scanning continues. Since the CT-simulator and RPM system are separate devices, they must be synchronised in the temporal space. A “scanner on” signal is sent to the RPM computer only when the CT-simulator is performing the scanning. Once the entire scan is complete, this signal and the breathing cycle are stored in a file and along with the CT images are exported to the GE AdvantageSIM workstation. At the AdvantageSIM workstation, the images are sorted according to their phases in a series of three-dimensional (3D) images, each representing a different phase of the respiratory cycle.

After the phases are chosen for gating, the image set of the end-expiration phase and all phases within the gating window are forwarded to the treatment planning system for the generation of a treatment plan. The end-expiration phase image set is chosen for contouring and planning because it represents the phase where the anatomical structures typically move the least for the longest time. On the respiratory cycle, it is depicted as a relatively “flat” signal at the minimum position (end-expiration phase) on Figure 2. However, neighbouring phases within the gating window can correspond to a time where the target and organs begin to move rapidly. Planning tools are often used to fuse images from different phases to determine the overall internal target volume (ITV). Contouring is done on the end-expiration phase image set but must include the location of the target and structure in all phases within the gate plus an additional margin for residual motion uncertainties. For lung targets where the background exhibits low Hounsfield (HU) numbers, all phases can be combined into a composite image. The maximum intensity projection (MIP) image constitutes a visualisation of the maximum extent of the target movement. Because of the large contrast between lung and target in the composite image, edge detection can be used to define the ITV, making contouring and planning simpler instead of image sets of all phases within the gate.

For respiratory-gated radiation therapy, the RPM system must be available at the treatment machine. The patient setup would be the same and the patient is coached in the same manner as during 4DCT scans. The respiratory-gated treatment technique is mechanically invoked prior to initiating treatment. The radiation therapists (radiographers) should monitor the respiratory cycle very carefully during dose delivery since the periodicity of the motion can change if the patient fails to follow the coaching instructions possibly resulting in inaccurate treatment. Predictive filters can be enabled in the software to limit this possibility.

**Figure 4:**
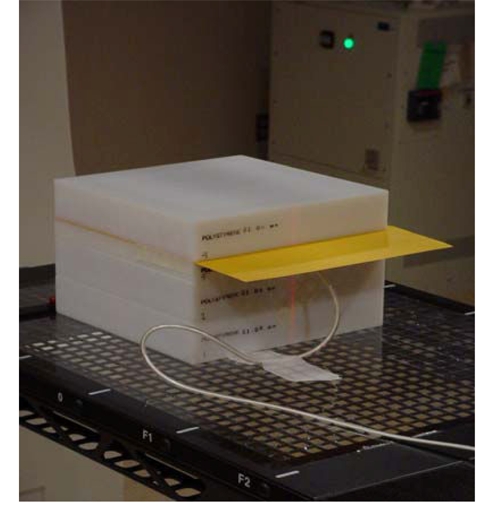
Phantom setup to perform patient specific dose validation. [With permission from C.B. Saw Publishing].

Individualised respiratory-gated IMRT treatment plans must also be validated prior to patient treatment. The validation procedure involves irradiating a phantom using individualised patient treatment parameters. The phantom setup used at this author’s institution is shown in Figure 4 with an ion chamber positioned at the isocenter and an EDR2 film positioned in a plane 1 cm above the isocenter. The monitor units, field geometries, fluence maps, and leaf sequences used for irradiation is the same as those used to treat the patient. The planning system is used to generate a hybrid test plan matching this validation delivered to the phantom for comparison purposes. After the dose delivery, the measured dose using the ion chamber is compared to the planned dose. In addition, the film is developed and the isodose distribution is measured using film dosimetry and compared to the isodose distribution in that plane as planned.

## RESULTS

**Figure 5:**
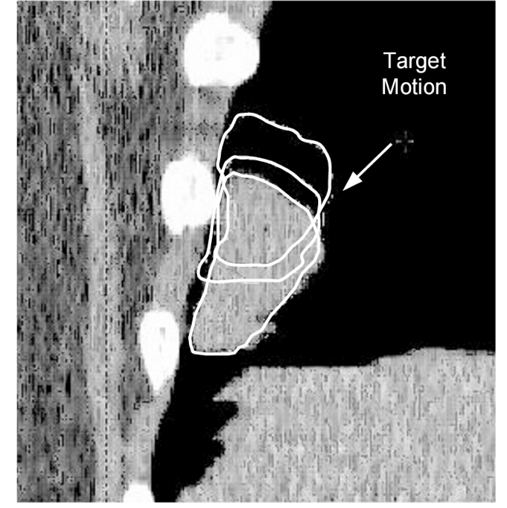
The relative motion of the target at three different phases.

After completing the retrospective 4DCT scans, the motion of the target in each respiratory phase can be traced and evaluated throughout the respiratory cycle. Since the CT images are acquired sequentially, the time resolution of the images represents the time difference between the phases. The superposition of images from different phases provides an idea of the relative motion of the target as shown in Figure 5. If respiratory-gated radiation therapy is selected, the number of phases used forms the gating window size with a defined residual tumour motion. The residual tumour motion (maximum motion of the target within the treatment field) has been analysed in phantom studies and a motion of up to 5 mm was found to be acceptable for the respiratory-gated IMRT treatment technique [18]. With this criterion, the end-expiration phase and its neighbouring phases are selected such that the target motion is kept to less than 5 mm. As many phases as possible should be included in the gating window to reduce the treatment time (i.e. maximising the duty cycle). If all the phases are included, it indicates gating is not needed. Based on this author’s experience, an approximate phase range of 30%-70% with a residual target motion of 5 mm within the gate is common.

Brandner *et al*. [14] at the University of Pittsburgh Cancer Institute has investigated the motion of some organs using the 4DCT scanning technique. They found that the organ motion is typically in the inferior-superior direction with an average of 12-15 mm for the kidneys, 15 mm for the liver and spleen, and 10 mm for the pancreas. Very little motion was seen in the left and right direction typically less than 3 mm for abdominal organs. In addition, the actual movement of the organs may span two or three dimensions indicating that organs must be evaluated in three dimensions on all respiratory phases. They also note that the superior lobe tumours move much less than inferior lobe tumours.

**Figure 6:**
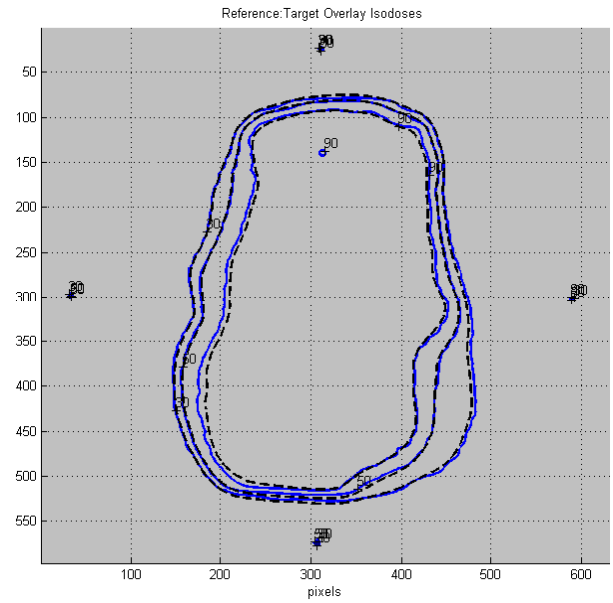
Measured and planned isodose distributions of a typical IMRT plan. [With permission from C.B. Saw Publishing]

The intent of respiratory-gated IMRT plan validation is to verify that the correct dose and dose distribution will be delivered to the patient. Generally, a point dose measurement is taken with an ion chamber and the difference between the measured dose and the calculated dose should be within 5%. In addition, the dose distribution in a plane is measured and compared to the treatment plan as shown in Figure 6. At this author’s institution, the difference in isodose levels of individualised treatment plan should be within 3 mm or 3% in high-dose region and with 5 mm and 5% in low-dose region to be considered acceptable.

**Figure 7:**
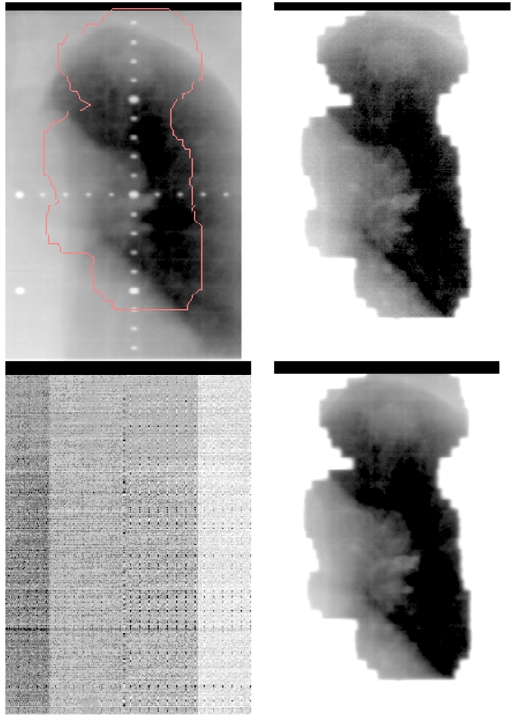
Comparison between DRR and portal images of a gated treatment

Figure 7 illustrates the effectiveness of implementing the respiratory-gated treatment technique. The upper left image of the figure is the digitally reconstructed radiograph (DRR) and the upper right image is the portal image acquired during the treatment. The lower left represents an image when the patient’s respiration was outside the respiratory gate and therefore the image is blurred since it was acquired while the beam was held. The lower right image was acquired when the beam came back on within the gate. Since the structures within the ports are observed not to move relative to the DRR images, it demonstrates the accuracy of the respiratory-gated treatment technique for radiation therapy.

## DISCUSSION

For the purpose of discussion, it should be pointed out that there is no basis for establishing the correlation between respiratory motion based on an external marker and the movement of the target. First, the dimension of the system is different. The respiratory motion is inferred based on the detection and recording of the marker block movement in the anterior-posterior dimension. On the other hand, the target or tumour, if mobile, can move in three dimensions in a complicated fashion. Second, it is well known that fixed tumours such as those fixed to the rib cage may not move in any synchronised fashion with the respiratory motion. Third, the work of Brandner *et al*. [14] has demonstrated that tumours in the lung move at different rates depending on their locations within the lung. However, the respiratory-gated treatment technique can be employed for a subset of patients determined using 4DCT scans where the target movement relative to the respiratory cycle is observed and assumed to be correlated to the respiratory cycle.

The 4DCT scanning technique allows a mechanism of establishing the relationship between the phases of the respiratory cycle, the gating window, and the tumour residual motion. If the gate is opened completely, implying that there is no gating, the tumour residual motion can be very large. This type of radiation treatment would require large margins resulting in the use of large treatment fields. If the gate can be restricted, the tumour residual motion would be smaller. This means that the radiation beam is turned ON within the gate as the target resides within the treatment field. If the tumour residual motion is further limited for an even smaller margin, the gate has to be made very small and thereby decreasing the duty cycle. The duty cycle is the ratio of the beam-on treatment time to the total treatment time. The objective of the respiratory-gated treatment technique is to search a means of increasing the gating window as large as possible for a defined (or smaller) tumour residual motion specification.

By its very nature, the respiratory-gated treatment technique accounts for the positional change with time and hence positional quality assurance is important. The performance of a positional check involves taking respiratory-gated CT images of a motion phantom from which to create the digital reconstructed radiographs (DRRs). The same phantom is then set up in the treatment room from which respiratory-gated portal images are acquired. The comparison of the respiratory-gated DRRs and respiratory-gated portal images provide estimates of the geometric uncertainty of the treatment delivery system under ideal conditions. It should be noted that this quality assurance does not address the issue of patient setup and variability of patient breathing motion during the course of treatment. The dosimetric errors associated with patient setup and breathing motion variability have been discussed in the report published by AAPM Task Group 76 [19]. In addition to positional confirmation, the dosimetry quality assurance involves measuring dose delivered to a moving phantom using a gated plan and comparing this to the dose delivered to the phantom while stationary. This provides assurance that the machine output is basically same with and without gating. The introduction of the respiratory-gated treatment technique has produced the risk of mistreatment where the radiation therapist (radiographer) may fail to invoke the respiratory-gated technique, invoking the respiratory-gated technique when not prescribed, or treating another patient with a different patient’s respiratory-gated parameters. Hence, radiation therapists (radiographers) must be more vigilant in reviewing the additional gating instructions and observing the respiratory trace.

## SUMMARY

To summarise, the respiratory-gated treatment technique using an external marker can be successfully implemented on selected patients. A requirement for the successful use of the respiratory-gated treatment technique is the need for the respiratory cycle to be periodic, reproducible, and maintained during treatment. This may not be possible for all patients especially those patients with compromised pulmonary function. Coaching should be used to assist in producing and maintaining the periodicity of the respiratory signals. The respiratory-gated treatment technique is ideal for lung and gastrointestinal cancer patients since its utilisation may potentially reduce radiation damage to surrounding normal tissues. However, the prerequisites require cautious assessment of respiratory motion in these patients. The movement of the target or adjacent organ(s) must be correlated to the respiratory cycle. The gating window should be reasonably large based on a specified tumour residual motion to maximise the duty cycle. 4DCT scanning is performed to evaluate and identify those patients that may benefit from this advanced radiation treatment technique. If the respiratory-gated treatment technique is employed, the treatment planning is performed on the end-expiration image set or a MIP image set that include all treatment phases. Contouring includes the tumour residual motion and an additional margin to allow for minor deviation from the respiratory cycle. Lastly, the respiratory-gated IMRT plans must also be validated prior to patient treatment to ensure that the doses are delivered in accordance to the treatment plan. Since respiratory-gated treatment technique is only used for selected patients and it is an add-on device, radiation therapists (or radiographers) are advised to be aware of invoking the gating technique when prescribed and be vigilant in observing additional parameters during treatment. Quality assurance aspects on the respiratory-gated treatment technique must address the positional and output change with time requiring a specialised motion-dependent phantom.
